# Natural and Synthetic Progestins Increase Transcriptional Expression of primiR-190 and primiR-199 in T47D Breast Cancer Cells: A Preliminary Study

**DOI:** 10.7759/cureus.78293

**Published:** 2025-01-31

**Authors:** Isabella Porter, Hannah E Berko, Benford Mafuvadze

**Affiliations:** 1 Anatomy and Molecular Medicine, Alabama College of Osteopathic Medicine, Dothan, USA

**Keywords:** breast cancer, primir-190, primir-199, progestins, pr-positive breast cancer

## Abstract

Background

Previous studies have shown that aberrant expression of different microRNAs potentially contributes to carcinogenesis, growth, and metastasis of several human cancers. Given that progestins have been reported to alter the expression of microRNAs in various human cancers, we hypothesized that progestins potentially influence the growth of hormone-responsive breast cancer through mechanisms involving the regulation of miRNAs functioning either as tumor suppressors or oncogenes. Using computer-based analysis, we identified two microRNAs that we investigated in this study, namely miR-190 and miR-199. Our main objective in this preliminary study was to determine the effect of different progestins on the expression of these two microRNAs in breast cancer cells.

Methods

Progesterone receptor (PR)-positive cell line, T47D breast cancer cells were exposed to progesterone and three different synthetic progestins for 24 hours, after which RNA was extracted and real-time polymerase chain reaction (PCR) was used to determine the expression of primiR-190 and primiR-199. For comparison, progestin effects were also tested in T47Dco-Y, a PR-negative cell line.

Results

Our results showed exposing T47D cells to both progesterone and synthetic progestins increased the transcriptional expression of primiR-190 and primiR-199a1 by as high as four to seven fold (P<0.0001). RU-486, a progesterone receptor antagonist, suppressed progestin induction of both primiR-190 and primiR-199a1. Progestin-induced effects were not observed in a PR-negative subline of T47D cells (P>0.05), further confirming the involvement of progesterone receptor-dependent pathways. Additionally, 17β estradiol and dimethyl sulfoxide did not alter the expression of both primiR-190 and primiR-199a1.

Conclusion

Different progestins increase transcriptional expression of both primiR-190 and primiR-199a-1 through progesterone receptor (PR)-dependent mechanisms. Both primiR-190 and primiR-199a1 can potentially be useful as biomarkers for PR-positive breast cancer.

## Introduction

Breast cancer is the most diagnosed cancer in women worldwide, with nearly 70-80% of newly diagnosed breast cancer being hormone sensitive and stimulated to grow by estrogens and progestins [1-3]. Despite clear evidence that shows an increased risk of breast cancer recurrence and metastasis in postmenopausal women taking combined estrogen and progestin hormone replacement therapy (HRT) [4-6], the molecular mechanism for progestin effects is still not fully understood. In general, progestins have been characterized to exert their genomic effects in breast cancer cells and other tissues through binding to the intracellular progesterone receptor (PR), a ligand-dependent transcription factor that is dimerized and translocated to the nucleus upon activation. Within the nucleus, the dimerized PR recruits coactivators and chromatin-remodeling complexes to activate the transcription of progesterone-responsive genes, which modulates the bioavailability of molecules needed for tumor growth [7]. For example, several studies have shown that exposure to progestins significantly alters the expression of factors like vascular endothelial growth factor (VEGF) [8-10] and CD44v6, which functions as a co-receptor to VEGF [11], which promotes tumor vascularization. Given that in a study by Goyette et al. [11], increased membrane expression of CD44v6 occurred with no evidence of direct binding of PR to the CD44 gene promoter, we hypothesized that a possible mechanism could involve progestin-induced alteration of key microRNAs.

Several studies in recent years have shown that aberrant expression of specific microRNAs occurs in different types of human cancers [12]. MicroRNAs are small non-coding RNAs that post-transcriptionally regulate gene expression and can control a broad spectrum of normal and pathological cellular functions [13-15]. MicroRNAs can act either as tumor suppressors or oncogenes and as such, a decrease or increase in specific microRNAs can profoundly affect cancer growth [15]. Changes in the expression of specific microRNAs have been observed at different stages of cancer development and may be involved in molecular mechanisms promoting the growth, progression, and metastasis of different cancers [16]. Because cumulative evidence shows that progestins can alter the expression of various microRNAs in cancer cells and other tissues [17], we initially used computer-based analysis to identify microRNAs with potential binding sites on known downstream targets for progestins. Our analysis identified the two miRNAs that we focused on in this study, namely, miR-190a-3p/5p and miR-199a-3p/5p. MicroRNAs are initially synthesized as long strands, which are then processed through enzyme-catalyzed cleavages that give rise to the mature functional forms of the microRNA [14,15]. In this study, we determined the expression of unprocessed microRNA strands (priMiRs) in PR-positive breast cancer cells exposed to different progestins. 

Based on our computer-based analysis, the first microRNA we investigated was primiR-199, whose mature form, miR-199a-3p, was revealed to have potential binding sites on known progestin genomic targets such as CD44 and associated regulatory proteins such as ZEB1, ESRP1, HIF-1a, and VEGF. In humans, the microRNA-199 family is comprised of primiR-199a1, primiR-199a2, and primiR-199b, encoded within introns of the dynamin genes in three separate loci: chromosome 19, where it is embedded in the anti-sense strand of intron 15 of Dynamin 2 (miR-199a-1); chromosome 1, where it is embedded in the anti-sense strand of intron 14 of Dynamin 3 (miR-199a-2); and DNM1 in chromosome 9 (primiR-199b) [18]. Many studies have shown that the mature forms of miR-199a, miR-199a-3p, and miR-199a-5p can regulate both proliferation and angiogenesis in cancer [19]. Some cancers, like gastric cancer, and miR-199a reportedly promoted cancer progression, and high expression of miR-199a correlated with poor patient prognosis [20]. The second microRNA we investigated in this study, miR-190a-3p/5p, was not only predicted to have a direct binding target for CD44 and VEGF but was also predicted to have a direct binding target on the progesterone receptor (PR) gene (www.mirdb.org). Binding to the progesterone receptor gene could provide an alternative mechanism through which miR-190a-3p/5p could directly regulate PR expression and, subsequently, progestin-dependent effects in breast cancer cells. In humans, miR-190 is encoded within the proximal end of the long arm of chromosome 15 and has two main mature forms: miR-190-5p and miR-190-3p [12]. MicroRNA-190-5p has been reported to function as both a tumor suppressor and oncogene in multiple human cancers, with its upregulation reported in pancreatic cancer [21] and bladder cancer [22], whereas downregulation was found in some forms of breast cancer [23], prostate cancer [24], and cervical cancer [25]. Interestingly, clinical data suggest that miR-190-5p may play a dual role in carcinogenesis and tumor progression, with some studies showing that miR-190-5p suppresses cell migration, invasion, and epithelial-to-mesenchymal transition (EMT) transformation in some breast cancers. In contrast, some studies reported that miR-190-5p promoted metastasis in some cell lines [23]. Thus, it is essential for us to elucidate specific effects of microRNA in different tumors and at various stages of tumor development.

It is clear that the role of progestins in breast cancer development is complex and may depend on several factors, including developmental stage as well as levels of estrogen present at the same time [26]. While there are apparent differences in microRNA profiles between hormone-responsive and triple-negative breast cancer [27], the effects of progestins on microRNA expression in breast cancer remain underexplored. Not many studies have looked at the effects of different progestins on the expression of primiR-190 and primiR-199a1. This study sought to address the gaps in knowledge by investigating the effects of exposing progesterone receptor (PR)-positive human breast cancer cells to different progestins on the transcriptional expression of primiR-190 and primiR-199a1. Our results demonstrate that both natural and synthetic progestins significantly increase the transcriptional expression of primiR-190 and primiR-199a1 in T47D breast cancer cells.

## Materials and methods

The study was conducted at the Department of Anatomy and Molecular Medicine, Alabama College of Osteopathic Medicine, Dothan, Alabama, USA.

Identification of miR-190 and miR-199 as microRNAs of interest

We used computer software analysis to identify miRNAs that directly target two previously identified progestin-induced genomic targets, namely, human VEGFA and CD44. Target prediction was done using two main databases: www.mirdb.org and www.targetscan.org. We narrowed our searches to only miRNAs with a target score between 50 and 100. Our results identified 239 potential miRNAs for CD44, including miR-190a-3p (100) and miR-199a-3p (78).

Our analysis predicted 232 potential targets for VEGFA, with miR-190a-3p showing a target score of 100. MiR-199a-3p was predicted to have a binding site in the conserved region of CD44 (www.targetscan.org), which we confirmed to have a target score of 78 (www.mirdb.org). We next investigated whether these identified miRNAs also had predicted binding sites on genes of proteins known to be involved in the regulation of CD44, VEGF, and associated angiogenic factors previously identified as induced by progestins. Our analysis revealed that in addition to a predicted binding site for CD44 and VEGF, miR-199a-3p/5p was also predicted to bind to genes for proteins known to regulate CD44 expression, such as ESRP1, ZEB1, and HIF-1a, while miR-190a-3p/5p showed predicted binding sites for ZEB2. Interestingly, miR-190a-3p/5p had a predicted binding site on the progesterone receptor (PR) gene. This study, thus, focused on determining the effects of exposing breast cancer cells to different progestins on the expression of primiR-190 and primiR-199. 

Cell culture and treatments

Progesterone receptor-positive (PR+) breast cancer cell lines, T47D and BT-474, were acquired from the American Type Culture Collection (ATCC). T47-Dco-Y cells, which are progesterone-receptor-negative clone derivatives of T47D cells, were kindly provided by Dr. Salman Hyder, University of Missouri. All cells were maintained and grown at 37°C in Dulbecco’s Modified Eagle’s Medium (DMEM)/F12 medium (Cat#: 11320082, Thermo Fisher Scientific Inc., Waltham, MA, USA) supplemented with 10% fetal bovine serum (Cat#: 12103C-100ML, Sigma-Aldrich, Co., St. Louis, MA, USA) in a humidified atmosphere of 5% CO2. In general, before exposure to the different progestins, cells were treated with DMEM/F12 supplemented with 5% dextran-coated charcoal (DCC)-treated FBS (Cat#: F6765-500ML, Sigma-Aldrich, Co.) for 24 hours. Subsequently, cells were washed with phosphate-buffered saline (PBS) prior to treatment with different progestins. Treatments with 1 µM RU-486 (Cat #: 475838-50 mg, Sigma-Aldrich, Co.) were always done at least two hours prior to the addition of specific progestins. Synthetic progestins tested in this study included medroxyprogesterone acetate (MPA, 10 nM, 1 nM, cat #: M1629-1G, Sigma-Aldrich, Co.), norgestrel (Norg, 10 nM, cat #: N2260, Sigma-Aldrich, Co.), and norethindrone (NorE, 10 nM, cat #: PHR1714-1G, Sigma-Aldrich, Co.). Other compounds also tested included the natural hormone, progesterone (P, 10 nM, Cat #: P8783-25G, Sigma-Aldrich, Co.), 17β estradiol (E2, 10 nM, Cat#: E8875-250MG, Sigma-Aldrich, Co.), and dimethyl sulfoxide (DMSO, Cat #: SC-358801, Santa Cruz Biotechnology Inc., Dallas, TX, USA). The concentrations of the different progestins used in this study have been used in previous studies [8-11] and confirmed to elicit progestin-induced changes in transcription of several genes in T47D cells within 24 hours. In all experiments, control cells were treated with an equivalent volume of ethanol, the vehicle media used to dissolve the different progestins.

RNA extraction and real-time polymerase chain reaction (PCR)

Following exposure to the different progestins for at least 20-24 hours, total RNA was extracted using a spin column RNA isolation kit (Cat#: 12183018A, Thermo Fisher Scientific Inc.) according to the manufacturer’s instructions. About 600 ng of extracted RNA was reverse-transcribed into cDNA using a high-capacity DNA synthesis kit and random primers (Cat#: 43-688-14, Applied Biosystems, Thermo Fisher Scientific Inc.). The synthesized cDNA was then amplified using a real-time PCR instrument (Applied Biosystems, Thermo Fisher Scientific Inc.), specific TaqMan primers, and TaqMan® Universal PCR Master Mix. Taqman Fam probes for human primiR-190 (hsa-miR-190, Cat#: HS03303305_pri, Thermo Fisher Scientific Inc.) and primiR-199 (hsa-miR-199a-1, Cat #: HS03302808_pri, Thermo Fisher Scientific Inc.) were used. Glyceraldehyde-3-phosphate dehydrogenase (GAPDH) Vic (Cat#: HS03929097, Thermo Fisher Scientific Inc.) was used as the endogenous control for all experiments. Relative gene expression was then determined using the following formula: Fold-change in gene expression; 2-ΔΔCt = 2 - {ΔCt (treated samples) - ΔCt (untreated control samples)}, where ΔCt = Ct (Target miR) - Ct (GAPDH), where Ct represents threshold cycle number. All real-time PCR reactions were carried out in duplicate, and at least two independent experiments were performed.

Statistical analysis

Data were reported as mean ± standard error of the mean (SEM). Statistical significance was tested by one-way analysis of variance (ANOVA) and Tukey’s multiple comparisons test for post-hoc testing using GraphPad Prism version 10.0.0 (131) software (Insight Venture Management, LLC, New York, US). Values of at least p < 0.05 were considered statistically significant.

## Results

Medroxyprogesterone acetate (MPA) increases transcriptional expression of primiR-190 and primiR-199 in T47D cells

**Figure 1:**
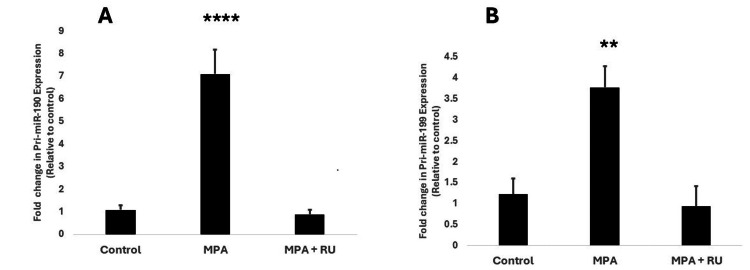
MPA increases primiR-190 and primiR-199 in T47D human breast cancer cells. T47D cells were treated at 37°C for 24 hours with 10 nM MPA, 10 nM + 1 µM RU-486 (RU), or an equivalent volume of ethanol (vehicle medium) for the control group. A: relative expression of pri-miR-190; B: relative expression of primiR-199a-1 was detected by real-time PCR. The levels of primiR-190 and primiR-199 were normalized to GAPDH, and the 2^-ΔΔCt^ method was used to determine fold change in expression relative to control. Bars represent mean ± SEM (n=5). **** Significantly different (ANOVA; P<0.0001), ** Significantly different (ANOVA; P<0.001). MPA: medroxyprogesterone acetate; PCR: polymerase chain reaction; GAPDH: glyceraldehyde-3-phosphate dehydrogenase

**Figure 2:**
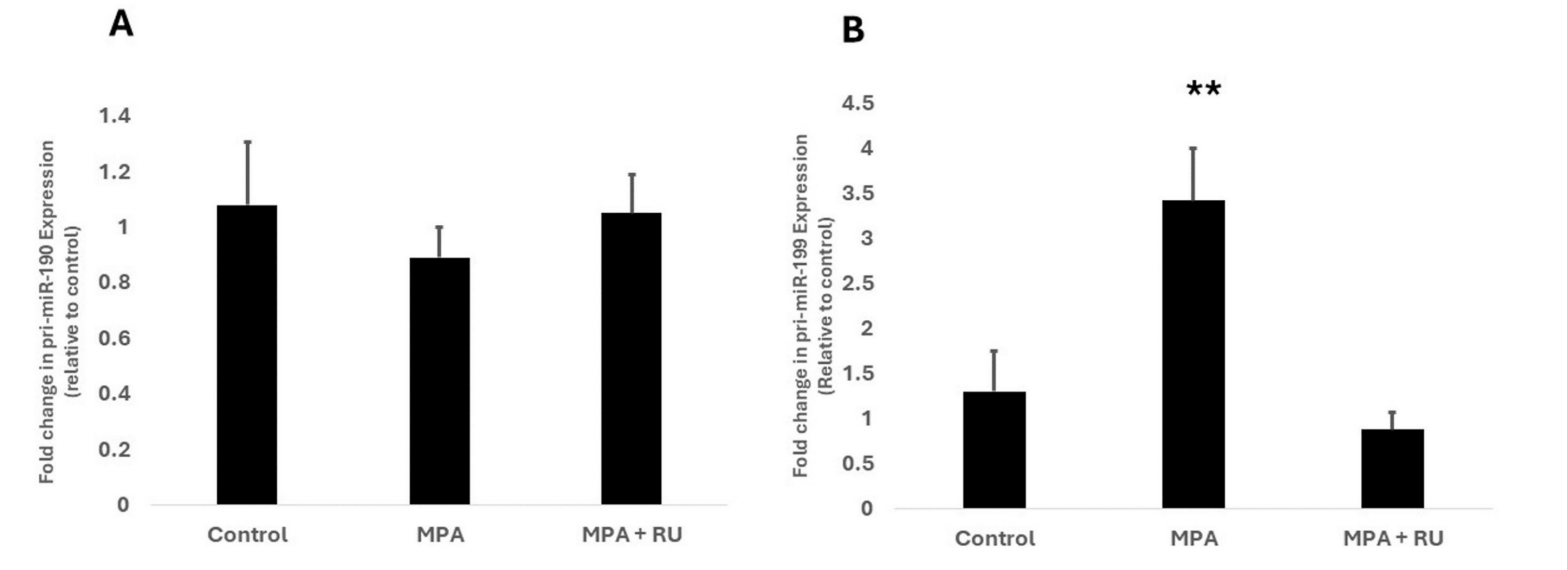
Effect of MPA on transcriptional expression of primiR-190 and primiR-199 in BT-474 human breast cancer cells. BT-474 cells were treated at 37^o^C for 24 hours with 10 nM MPA, 10 nM + 1 µM RU-486 (RU), or an equivalent volume of ethanol (vehicle medium) for the control group. A: relative expression of primiR-190; B: relative expression of primiR-199a-1 was detected by real-time PCR. The level of primiR-190 and primiR-199 were normalized to GAPDH and the 2^-ΔΔCt^ method was used to determine fold change in expression relative to control. Bars represent mean ± SEM (n =5). ** Significantly different (ANOVA; P<0.001). MPA: medroxyprogesterone acetate; PCR: polymerase chain reaction; GAPDH: glyceraldehyde-3-phosphate dehydrogenase

We initially sought to determine whether exposure to medroxyprogesterone acetate (MPA), a synthetic progestin widely used in the United States and other countries, affected the transcriptional expression of primiR-190 and primiR-199 in the progesterone receptor-positive (PR+) breast cancer cell line T47D. Our results showed that T47D cells exposed to MPA for 24 hours expressed nearly seven-fold higher levels of primiR-190 than control cells that received a similar volume of the vehicle medium, ethanol (Figure 1A). The expression of primiR-190 in the MPA + RU group was significantly lower (P<0.0001) than in the MPA alone-treated group and was not statistically different from the control group (p = 0.9781) (Figure 1A). Similarly, T47D cells exposed to MPA for 24 hours showed a four-fold higher expression of primiR-199 than control cells (Figure 1B). We further investigated the effects of MPA on primiR-190 and primiR-199 expression in another PR-positive cell line called BT-474. While exposure to MPA for 24 hours increased expression of primiR-199 in BT-474 more than three-fold higher as compared to the control group exposed only to the vehicle medium (P=0.014) (Figure 2B), we did not observe any statistically significant differences in expression of primiR-190 (Figure 2A).

Different progestins increase transcriptional expression of primiR-190 and primiR-199 in T47D cells

**Figure 3:**
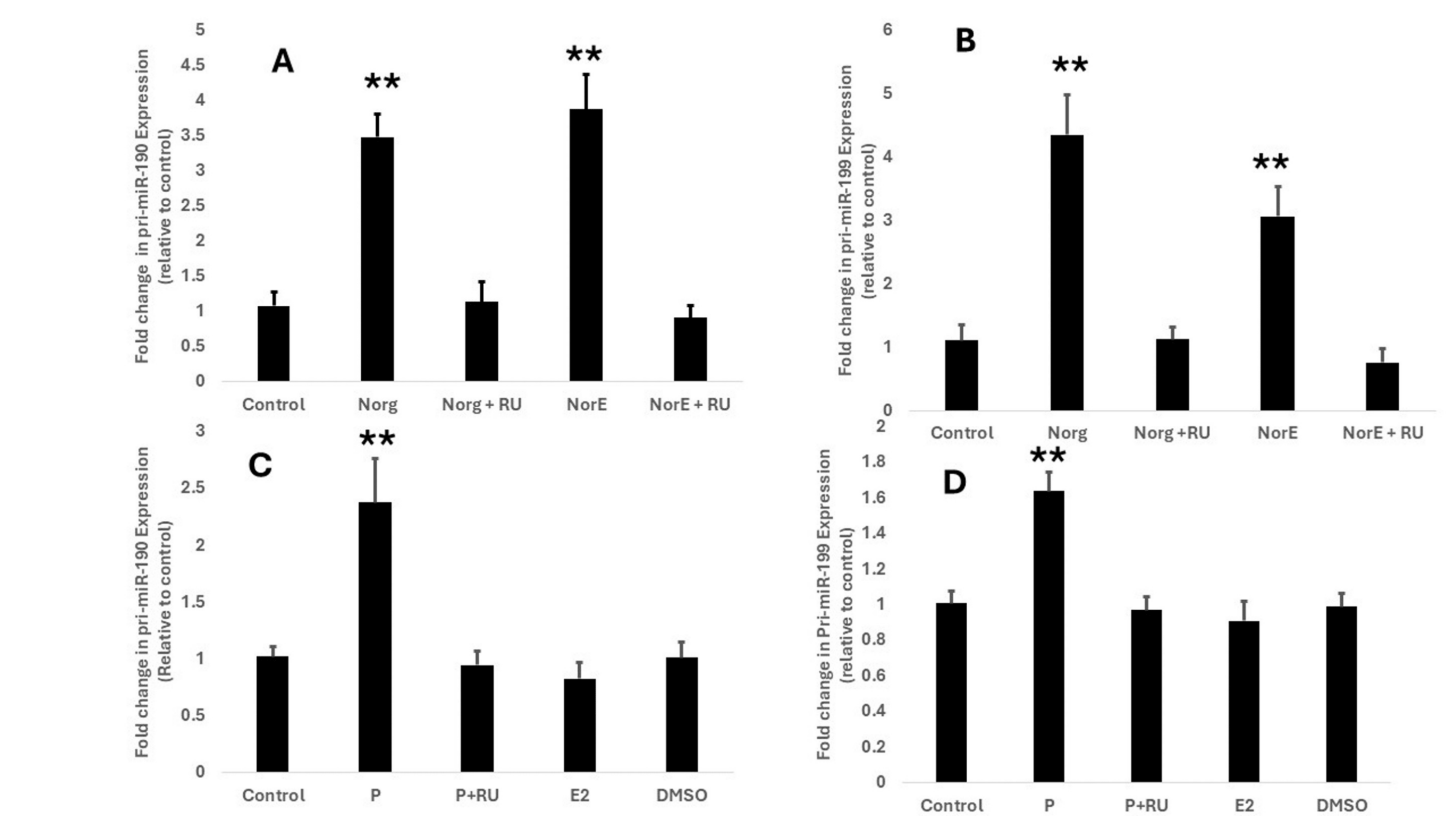
Effect of different synthetic progestins, progesterone, estradiol, and DMSO on the transcriptional expression of primiR-190 and primiR-199 expression. T47D cells were treated at 37^o^C for 24 hours with 10 nM norgesterel (Norg), 10 nM Norg + 1µM RU-486 (RU), 10 nM norethindrone (NorE), 10 nM NorE + 1µM RU-486, 10 nM progesterone (P), 10 nM P + 1 µM RU-486, 10 nM 17β-estradiol (E2), dimethyl sulfoxide (DMSO), equivalent volume of ethanol (vehicle medium) for control group. A: relative expression of pri-miR-190; B: relative expression of primiR-199a-1; C: relative expression of primiR-190; D: relative expression of primiR-199a-1. All microRNA levels were normalized to GAPDH and the 2^-ΔΔCt^ method was used to determine fold change in expression relative to control. Bars represent mean ± SEM (n =5). ** Significantly different (ANOVA; P<0.001). MPA: medroxyprogesterone acetate; PCR: polymerase chain reaction; GAPDH: glyceraldehyde-3-phosphate dehydrogenase

We set out to determine the effects of the natural hormone progesterone and other progestins on the expression of primiR-190 and primiR-199 transcriptional expression in T47D cells. Our results further showed that two synthetic progestins, norgestrel and norethindrone, significantly increased transcriptional expression of both primiR-190 (p<0.0001) and primiR-199 (p<0.0001) three- to four-fold higher as compared to the control group (Figures 3A, 3B). In addition, our results also showed that the natural hormone, progesterone, increased both primiR-190 (p=0.0015) and primiR-199 (p=0.0007) transcriptional expression as compared to control groups (Figures 3C, 3D). In all experiments, the effects of progesterone, norgestrel, and norethindrone were significantly suppressed when cells were exposed to the progesterone receptor (PR) antagonist RU486 (mifepristone) two hours prior to progestins administered, resulting in the expression of both primiR-190 and primiR-199 not statistically different from the control group (Figures 3C, 3D). These results suggested that the progestin-induced increase in expression of primiR-190 and primiR-199 occurred through PR-dependent pathways. Given that RU486 is not exclusively selective for PR and is also known to block the effects of other steroid hormones, we sought to determine whether other steroids like 17B-estradiol could also alter the transcriptional expression of these microRNAs. In previous studies, estradiol has been shown to have opposing effects to progestins in some human cancers [28]. In this study, exposing T47D cells to both 17B-estradiol and DMSO for 24h had no significant effect on the expression of both primiR-190 and primiR-199 (Figures 3C, 3D).

Progestins had no effect on primiR-190 and primiR-199 expression in T47Dco-Y cells

**Figure 4:**
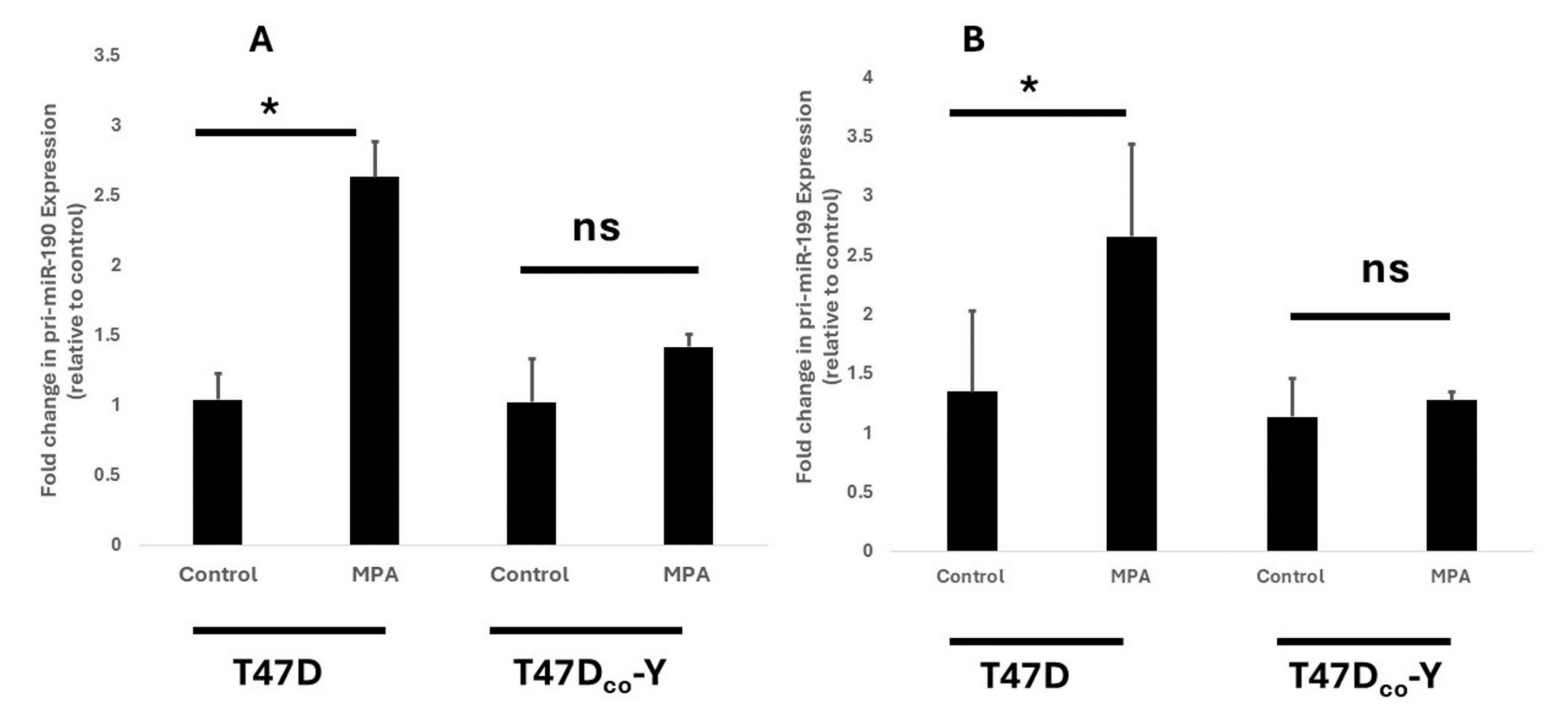
Effect of MPA on primiR-190 and primiR-199a-1 transcriptional expression in PR-negative T47Dco-Y cells. T47D (PR-positive) and T47Dco-Y (PR-negative) cells were treated at 37^o^C for 24 hours with 1 nM MPA or an equivalent volume of ethanol for the control group. A: relative expression of primiR-190; B: relative expression of primiR-199a-1 was detected by real-time PCR. The level of primiR-190 and primiR-199 were normalized to GAPDH and the 2^-ΔΔCt^ method was used to determine fold change in expression relative to control. Bars represent mean ± SEM (n =5). * Significantly different (ANOVA; P<0.05, ns; no significant difference). MPA: medroxyprogesterone acetate; PCR: polymerase chain reaction; GAPDH: glyceraldehyde-3-phosphate dehydrogenase

Given that our previous studies suggested that exposure to progestins produced a significant increase in transcriptional expression of both primiR-190 and primiR-199 through mechanisms mediated through the progesterone receptor (PR), we examined the effects of both MPA and progesterone in T47Dco-Y, a stable PR-negative monoclonal subline of the PR-positive T47D cell line. We hypothesized that knocking down PR receptors would significantly reduce progestin-induced transcriptional expression of both primiR-190 and primiR-199a-1. Our results show that both MPA (Figures 4A, 4B) and progesterone (unshown data) failed to increase the transcriptional expression of both primiR-190 and primiR-199 in T47Dco-Y. At the same time, there was a significant increase in T47D cells simultaneously exposed to the progestins.

## Discussion

Cumulative evidence shows that microRNAs are frequently dysregulated in human breast cancers, where they act either as oncogenes or tumor suppressors and contribute significantly to the development and progression of these cancers [14]. Given that nearly 70-80% of human breast cancers are hormone-responsive, it is important that we explore how hormones like progesterone and synthetic progestins may be involved in the regulation of microRNAs that may be involved in the carcinogenesis and metastasis of these cancers. Previous studies have described different mechanisms through which progestins promote cancer development, including stimulation of tumor angiogenesis through the induction of factors such as VEGF [8-10] and CD44v6 [11]. In a study on CD44 expression, Goyette et al. [11] showed that PR did not bind directly to the CD44 promoter to influence transcription or splicing, which made us hypothesize that observed genomic changes possibly occurred through progestin-induced alteration in the expression of specific microRNAs. We, thus, performed a computer-based analysis that identified two microRNAs with potential binding sites for well-known progestin-regulated genomic targets such as CD44 and VEGF, namely, miR-190a-3p/5p and miR-199a-3p/5p. Previous studies revealed some clinical associations between microRNA-190 and the occurrence of different human cancers. For example, upregulation of the mature form of primiR-190, miR-190-5p, was reported in pancreatic cancer, bladder cancer, meningioma, and gastric cancer [12]. In contrast, downregulation was reported in some breast cancer, hepatocellular carcinoma, rectal cancer, cervical, and prostate cancer [12]. Our computer analysis also identified miR-190a-3p/5p as having a direct binding target for the progesterone receptor (PR) gene, suggesting it could potentially regulate progestin-dependent effects in breast cancer through a direct regulation of PR.

Our first goal in this preliminary study was, thus, to determine the effects of exposing PR-positive breast cancer cells to both natural and synthetic progestins on transcriptional expression of primiR-190. Our results showed that progesterone and three different synthetic progestins (Figures 1A, 3A, 3C) significantly increased transcriptional expression of pri-miR-190 in T47D cells. The progesterone receptor antagonist, RU486, blocked the effects of all progestins on pri-miR-190 expression. On the other hand, our study showed that both MPA (Figure 2A) and progesterone (data not shown) did not significantly affect primiR-190 expression in another PR-positive breast cancer cell line, BT-474 cells. These findings from our study appear to show similar patterns to what was reported by Goyette et al. [11] in a study examining the effects of progestins on CD44v6 expression where differences between T47D and BT-474 cells were observed. While both T47D cells and BT-474 express progesterone receptors, the main difference between them is that T47D cells express significantly more progesterone receptors than BT-474 cells, while BT-474 cells express significantly more HER2. Our results suggest that the mere presence of progesterone receptors is not adequate, and additional factors are potentially involved in progestin-dependent regulation of primiR-190 expression. For example, it is possible that there is crosstalk between PR and other signaling pathways, such as those involving the estrogen receptor (ER) or growth factors, which could directly or indirectly influence the transcriptional activity of primiR-19. Alternatively, increases in primiR-190 expression could also be due to changes in its stability or processing efficiency. Hormonal signaling can sometimes influence factors involved in miRNA processing, such as Drosha and DGCR8 [14], which can impact the maturation rate and accumulation of specific primiRNAs. Identification and characterization of different molecular factors involved in the modulation of progestin-induced expression of microRNA-190 and other molecular markers such as CD44v6 [11] is crucial to fully understand the functional role of progestins in cancer development. It is also important that we perform studies correlating PR expression to the expression of miR-190 in human breast cancer biopsy tissues and correlate that to patient survival rates.

The next microRNA that we investigated in this study was microRNA-199a1. Previous studies have indicated the involvement of factors such as TWIST 1 and EGR1 in the regulation of miR-199a expression [18]. Our study adds to the growing list of factors known to regulate the expression of miR-199 in breast cancer cells by showing that both natural progesterone and synthetic progestins significantly increased transcriptional expression of primiR-199a1 in both T47D and BT-474 (Figures 1B, 2B, 3B, 3D). In an earlier study involving cultured human myometrial cells, Williams et al. [28] reported that progesterone increased miR-199a-3p expression while estradiol had the opposite effect. Our data showed that 17β-estradiol had no significant effect on expression of pri-miR-199a-1 with no difference observed between cells exposed to 17β-estradiol and the control group (Figure 3D). The lack of effect on the expression of both primiR-199 and primiR-190 with exposure to structurally similar steroids like 17β-estradiol provides further evidence that the observed increase in microRNA expression most likely occurs through mechanisms exclusively dependent on the classical intracellular progesterone receptor. These findings concur with previous studies that showed that progesterone regulation of key microRNA expression occurs via the classic nuclear PR pathways [29]. The failure to elicit progestin-stimulated increased expression of both primiR-199a1 and primiR-190 in a subline of T47D cells where progesterone receptors have been knocked out provides further support for the involvement of activated PR in the regulation of transcriptional expression of both microRNAs in breast cancer cells. Considering the well-established increase in the risk of metastasis and tumor recurrence in women taking combined estrogen and progestin hormone replacement therapy [4-6], there is a high probability that the progestin-induced increase in both primiR-199a1 and primiR-190 expression observed in this study may contribute to this increase in breast cancer invasiveness and metastatic potential. Several studies cited in the literature simply compared the expression of miR-199 in different breast cancer cell lines without exposing the cells to progestins. Our study provides evidence that exposing PR-positive breast cancer cells to progestin, as happens with women taking progestin-containing hormone replacement therapy, could tremendously increase expression of microRNAs in some breast cells that under normal physiological conditions would not express significant levels of these microRNAs, leading to altered expression of targeted genes. While our data suggest that the increase in both primiR-199 and primiR-190 may contribute to breast cancer invasiveness, some previous studies have linked a decrease in mature forms of miR-199 to increased invasiveness of some cancer cell lines [30]. Several microRNAs have been shown to have complex effects, with some showing dual effects, acting either as tumor suppressors or oncogenes in different cancers and tissues and at different stages of tumor growth [31]. It is possible that microRNA-199 and microRNA-190 may have different functional roles in breast cancers at different stages of tumor development.

This study is limited by the fact that we only tested two PR-positive breast cancer cell lines. Future studies should be directed at understanding the significance of the increase in transcriptional expression of both primiR-199 and primiR-190 observed in this study to determine the clinical implications on carcinogenesis, progression, and metastasis of human breast cancer cells and survival outcomes in patients. It would also be important to identify and characterize the key genomic targets affected by alteration in the expression of specific mature forms of these two microRNAs.

## Conclusions

This preliminary study provides evidence that different progestins significantly increase the transcriptional expression of primiR-190 and primiR-199 in some breast cancer cell lines. These findings suggest that both primiR-190 and primiR-199a1 might be useful as diagnostic and prognostic biomarkers for some PR-positive human breast cancers. More studies need to be done to fully understand the clinical implications of these two microRNAs on carcinogenesis, progression, and metastasis of hormone-responsive breast cancer, especially in women taking combined estrogen-progestin hormone replacement therapy.
